# Analyzing service descriptors and patients’ clinical characteristics may help understand heterogeneity in long-term trajectory of patients with schizophrenia, bipolar and major depressive disorder

**DOI:** 10.1371/journal.pmen.0000327

**Published:** 2025-05-14

**Authors:** Vittorio Nicoletta, Angel Ruiz, Valérie Bélanger, Thomas Paccalet, Maripier Isabelle, Michel Maziade

**Affiliations:** 1 Département opérations et systèmes de décision, Faculté des sciences de l’administration, Université Laval, Quebec City, QC, Canada; 2 Département de gestion des opérations et de la logistique, HEC Montreal, Montreal, QC, Canada; 3 Bureau de l’innovation et du numérique propulsé par la recherche (BIN^R^), Direction générale adjointe - Planification stratégique et performance, Centre intégré universitaire de santé et de services sociaux de la Capitale-Nationale, Quebec City, QC, Canada; 4 Département d’économique, Faculté des sciences sociales, Université Laval, Quebec City, QC, Canada; 5 Centre de recherche CERVO, Centre Intégré Universitaire de Santé et des Services Sociaux de la Capitale-Nationale, Québec, Canada; 6 Faculté de Médecine, Département de Psychiatrie et Neurosciences, Université Laval, Quebec City, QC, Canada; PLOS: Public Library of Science, UNITED KINGDOM OF GREAT BRITAIN AND NORTHERN IRELAND

## Abstract

One major obstacle to advancing research and treatment for major psychiatric disorders is their substantial within-diagnosis heterogeneity in patient lifetime trajectories. Adapted research methods such as cluster analysis to define subgroups of patients are currently used. However few studies have included service delivery descriptors in cluster analysis to investigate the determinants of heterogeneity in long-term trajectories. The aim of this study was to test whether patterns of service delivery could help in defining subgroups in terms of trajectories and clinical profiles in schizophrenia, bipolar disorder or major depressive disorder patients. Hierarchical Agglomerative Clustering (HAC) algorithms were used on a sample extracted from a Quebec government (Canada) transactional database to group and classify patients according to their interactions with the service delivery system. The resulting clusters were analyzed using statistical tools to characterize service trajectories. We observed three distinct trajectories that were not specific to any one of the three lifetime psychiatric diagnoses. Clusters were particularly affected by varying rates of clinician changes across the trajectory and changes of diagnoses. Results suggest that incorporating service delivery characteristics in future longitudinal studies of heterogeneity might be useful as a complement to studies that solely examine patients’ clinical features. The inclusion of service delivery elements may also be a useful tool for acquiring knowledge to adapt services to patients’ needs in public mental health and mental health economics research.

## Introduction

Schizophrenia (SZ), bipolar disorder (BD) and major depressive disorder (MDD) together affect more than 4% of the population, or about 35 million people in the G7 countries [1]. The majority of patients affected by SZ, BD or MDD follow a chronic course, mainly because treatments remain largely symptomatic [2,3]. One major obstacle to advancing research is the substantial within-diagnosis heterogeneity in trajectory profiles for major psychiatric disorders. Adapted research methods must be sought to advance knowledge about the sources of this heterogeneity and then improve treatment and service delivery. The relevance of longitudinal trajectory studies is also underscored by the reduced quality of life and productivity of patients, with economic costs that amount to hundreds of billions of dollars annually in Europe and North America [4–6].

Over the past decades, several heterogeneity studies have investigated the longitudinal course of major psychiatric disorders such as SZ, BD, and MDD. These studies are difficult to compare due to differences in methods and variables used. Many informative studies have examined governmental population-based data registries in different countries [7,8] suggesting that the degree of concordance between research diagnoses and clinician diagnoses is satisfactory [9–11].

Relatedly, several studies have raised concerns about the stability of diagnoses over time [12,13], an important issue that has not often been considered in cross-sectional studies and in studies of the course of major psychiatric disorders. Indeed, the heterogeneity of the long-term course of psychiatric disorders includes changes in diagnosis and symptom intensity that make it difficult to interpret for disease management and service delivery modalities. BD would show more diagnostic instability [14–16] than SZ [17–21].

The impact of changes in diagnosis over the course of a patient’s illness is poorly understood. From a clinical perspective, diagnosis changes over time may imply treatment modifications or clarifications for the patient and family, and even possible forensic implications.

From a research perspective, it can be inferred that diagnosis changes over time, if not accounted for in trajectory research, are likely to introduce biases in sampling and outcome interpretation. Finally, most trajectory studies have examined only one disorder, and very few have simultaneously examined the clinical trajectories of patients with SZ, BD, and MDD.

Four conclusions can be drawn from previous trajectory studies [7,19–23]. First, most have focused solely on demographic and disease characteristics such as gender, severity, age of onset, or cognitive changes [24,25]. Second, heterogeneity has been observed among patients with respect to their long-term course [7,26–28]. Third, greater severity in the early years of illness would predict a worse long-term course [26]. Fourth, as mentioned above, the few studies that have examined the degree of diagnostic change suggest a higher diagnostic instability in BD than in SZ or MDD [14–16].

Although some studies have attempted to examine heterogeneity in patients’ long-term trajectories by considering the three major lifetime psychiatric diagnoses [29–33], only a few have utilized clustering analysis to do so [34–36]. To our knowledge, no studies have explicitly incorporated service delivery descriptors in the analysis. Our objective was to assess the heterogeneity in the long-term trajectories of SZ, BD and MDD, by performing a cluster analysis on several service use descriptors and subsequently validating the obtained clusters using patients’ clinical characteristics. Our hypothesis was that service delivery patterns would contribute to interpretable heterogeneous subgroups of patient trajectories.

## Methods

We implemented a two-stage analysis process. In the first step, we extracted for each subject the number and type of patient contacts with health professionals from a government database containing health records for the entire population of Quebec City in the province of Quebec, Canada, from 2002 to 2014. The Quebec province offers a public universal health care system to its population. We then computed, for each subject, a set of service descriptors including the frequency of consultations, hospitalizations, change of professional, and diagnosis changes, and applied a cluster analysis to group individual trajectories into homogeneous groups. In a second step, analysis were performed on the resulting clusters to examine potential differences among the clusters in terms of patients’ clinical characteristics.

### Ethical approval

Data used in this research were provided by the Régie de l’assurance maladie du Québec (RAMQ), which is the Quebec government’s mandatory health insurance office. The RAMQ manages transactions related to the use of medical services and reimburses health care professionals in the province of Quebec, Canada. The access to anonymized and confidential RAMQ’s data was authorised by the Commission d’accès à l’information du Québec (Quebec Information Access Commission, CAI), the official governmental registry in the province of Quebec, under request Ref. 111031-S. Since the dataset provided by RAMQ was anonymized, individuals’ consents were not obtained. We are prohibited from sharing the data with any repository or publication. Conversely, anyone interested should address an information access request directly to the RAMQ’s (acces@ramq.gouv.qc.ca).

### Dataset

We had access to an anonymized RAMQ dataset containing all care services provided to individuals at all health centers in the Quebec City region between 2002 and 2014. This health administrative region covers more than 18,600 km^2^ and is populated by nearly 730,000 people from urban and semi-rural areas. We processed a dataset of 98 million observations corresponding to all patient transactions during the aforesaid period. Each record/transaction described a patient’s contact with the health care system and included the date and location of the contact, the anonymized identification number of the patient and the health care professional, and the associated principal diagnosis describing the encounter according to the diagnostic codes from the Ninth Revision of the International Statistical Classification of Diseases and Related Health Problems (ICD-09, see S12 Table) [37]. From this data set, we identified patients who met the following criteria: (1) received at least one ICD diagnosis of MDD, BD, or SZ; (2) received at least a diagnosis of MDD, BD, or SZ between the ages of 15 and 34 years; and (3) experienced a first contact with the system (considered here as a first episode) between 2002 and 2014. A total of 6,968 patients (3,752 men and 3,216 women) met these criteria.

### Service trajectories

**Fig 1:**
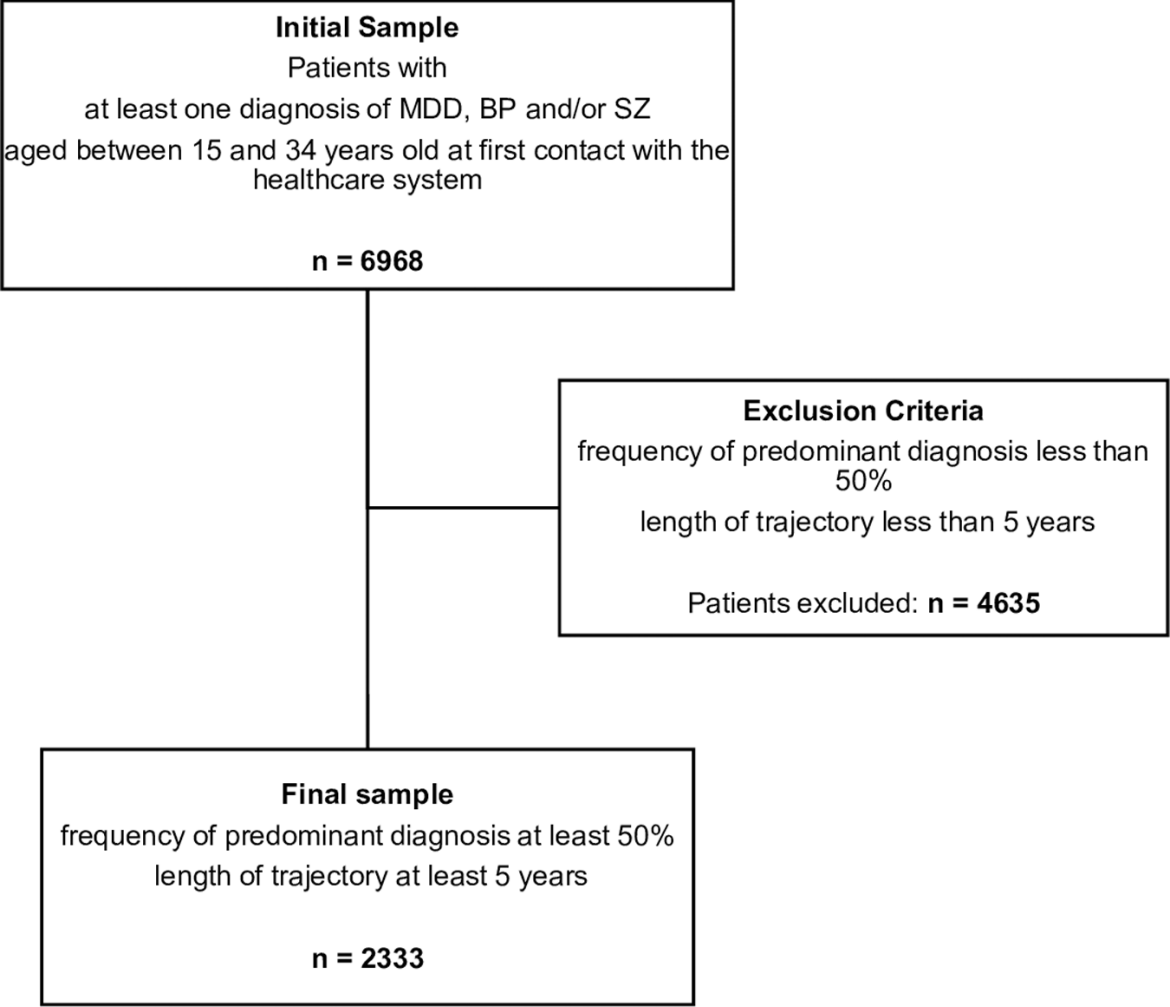
Steps to the final sample. The final sample includes 2,333 individuals.

For each patient, we extracted the records related to psychiatric services (consultations and hospitalizations). These contacts with services are hereafter referred to as “visits”. The chronological sequence of visits forms the patient’s service trajectory. We defined the span of the service trajectory as the interval between the patient’s first and last visits. To avoid the potential bias that individuals with sporadic visits might intrude into our sample, we excluded patients with less than five years of follow-up, resulting in a final sample of 2,333 individuals. **Fig 1** illustrates the step-by-step process of patient exclusion, whereas **Table 1** provides descriptive characteristics of the final sample.

**Table 1:** Demographic and clinical characteristics of patients in the final sample (N = 2 333).

Characteristics	Final sample N (%)
Total patients	2333
Male patients	1265 (54%)
Patients with first diagnosis of:	
Major Depressive Disorder	1014 (44%)
Bipolar Disorder	473 (20%)
Schizophrenia	846 (36%)
Patients with predominant diagnosis of:	
Major Depressive Disorder	759 (33%)
Bipolar Disorder	525 (22%)
Schizophrenia	1049 (45%)

### Diagnostic methods and predominant diagnosis along the trajectory

As reported in several previous studies of governmental health database [11,38,39], diagnoses were ICD diagnoses made by the clinicians across the trajectory of the patient.

A significant proportion of patients had changes of diagnosis given by the clinician over the course of the trajectory (see S1 Table). For this reason, a “predominant diagnosis” was necessary to obtain an equivalent of a lifetime diagnosis for each patient. We defined the predominant diagnosis as the diagnosis most frequently given by the clinician, i.e., the same diagnosis had to be present in at least 50% of the visits. If no diagnosis reached a frequency of at least 50% of the visits, the predominant (lifetime) diagnosis was considered uncertain, and the patient was excluded from further analyses.

### Measurements

The measures were related to the services provided and the disease characteristics of the patients. The five measures that assessed how patients interact with the care delivery system longitudinally (thus characterizing their service trajectories) were: 1. visit frequency, defined as the number of visits divided by the length of the trajectory; 2. median time between consecutive visits; 3. number of diagnosis changes, defined as the number of different diagnoses at two consecutive visits along the patient’s trajectory; 4. percentage of visits with a diagnosis change, defined as the number of diagnosis changes divided by the number of visits in the trajectory; 5. number of physician changes, defined as the number of times the diagnosis was made by different physicians. Patient disease characteristics were: 1. the patient’s sex; 2. the age at onset of illness (defined as the first contact with the health care system); 3. the predominant diagnosis along the trajectory; 4. the first diagnosis made at the first visit; and 5. the number of hospitalizations assumed if the patient had a series of visits within seven days or less.

### Statistical methods

First, we performed a cluster analysis on the patients’ service descriptors to identify subgroups of service trajectories [40]. We used the R statistical software (https://www.r-project.org/), which includes the k-means and hierarchical agglomerative clustering (HAC) methods. The k-means clustering is faster than the HAC, but it requires that the number of clusters be predetermined. Given the paucity of prior literature on our objective, we chose to test the number of clusters (or trajectories) that best fit our data, so we chose the HAC method. For the purposes of the analysis, the five measures of provider system characteristics were normalized to the [0,1] interval. Correlation tests between these measures resulted in low values of the R coefficient (less than |0.39| in all cases). The HAC algorithm first computes the distance between patients using the Euclidean distance; then it groups patients using a specific linkage function. Three linkage methods – Single, Complete, and Ward’s linkage – were evaluated, yielding agglomerative coefficient values of 97.03, 99.17, and 99.99, respectively. These results indicate a marginally higher clustering cohesion for the Ward’s linkage method. The correlation coefficients matrix and kernel density estimate for the five cluster variables are provided as supplementary material figures S1 Fig and S2 Fig. To select the appropriate number of classes, we first relied on visual inspection of the dendrogram, after which the Dunn index was calculated. A higher Dunn index was considered to indicate better clustering.

Second, the service trajectories within each class were analyzed to identify their common features, and descriptive statistics were computed to characterize the service trajectory subtype that fits each class.

Finally, regression analyses and ANOVAs tests using the clusters as dependent variables were performed to confirm the differences between the subtypes of trajectories identified by the cluster analysis (see subsection External validation of the clusters).

## Results and validation

### Service trajectory subtypes

**Fig 2:**
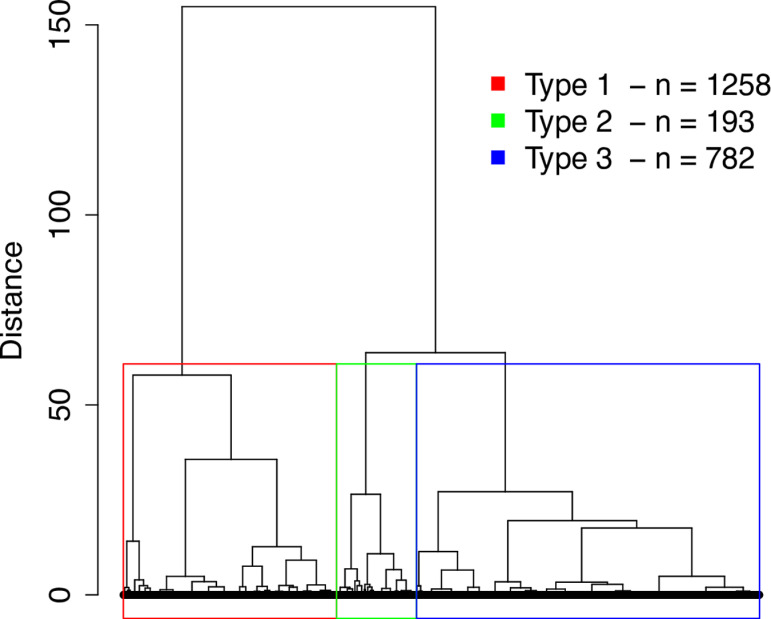
Dendrogram for trajectory analysis. Graphical representation of the three classes (Type 1, Type 2, and Type 3) as a dendrogram. Each of the 2333 patients is represented by a black dot at the bottom of the image and connected by vertical lines to the most similar patient. Groups of patients connected by shorter vertical lines are less distant and therefore similar. The three selected classes are highlighted.

A cluster analysis was applied to the service delivery descriptors of 2,333 individuals with a predominant diagnosis of MDD, BD, or SZ. The cluster analysis used the five measures that define patients’ interactions with the service provider: frequency of visits, median time between consecutive visits, number of diagnosis changes, percentage of visits with a diagnosis change, and number of times the diagnosis was made by different clinicians. The structure of classes or trajectory subtypes that best fit the data was based on the Dunn index, which yielded values of.007,.008,.008,.008, and.002 for structures with 2, 3, 4, 5, and 6 classes, respectively. The three-class structure was chosen on the principle that increasing the number of classes would not improve the Dunn Index value. The dendrogram is shown in **Fig 2**.

The description of the patients populating the three service trajectories or classes is given in **Table 2**.

**Table 2:** Characteristics of the three service trajectories or classes^a^.

	Final sample	Class 1	Class 2	Class 3
**Characteristics**	**N (%)**	**N (%)**	**N (%)**	**N (%)**
Total patients	2333	1258 (54%)	293 (13%)	782 (33%)
Male patients	1265 (54%)	730 (58%)	169 (58%)	366 (47%)
Patients with first diagnosis of:				
Major Depressive Disorder	1014 (44%)	510 (41%)	72 (25%)	432 (55%)
Bipolar Disorder	473 (20%)	189 (15%)	59 (20%)	225 (29%)
Schizophrenia	846 (36%)	559 (44%)	162 (55%)	125 (16%)
Patients with predominant diagnosis of:				
Major Depressive Disorder	759 (33%)	378 (30%)	20(7%)	361 (46%)
Bipolar Disorder	525 (22%)	204 (16%)	46 (16%)	275 (35%)
Schizophrenia	1049 (45%)	676 (54%)	227 (77%)	146 (19%)

^a^Class 1 refers to *Stable diagnosis* trajectory; Class 2 refers to *Unstable diagnosis with high care consumption* trajectory; Class 3 refers to *Intermediate unstable diagnosis with low consumption of care* trajectory.

The mean values of service indicators for the patients in each class, as well as their associated 95% confidence intervals, are given in **Table 3**.

**Table 3:** Average values and confidence intervals at 95% for the service measures in each trajectory or class^a^.

Characteristics	Final sample (CI 95%)	Class 1 (CI 95%)	Class 2 (CI 95%)	Class 3 (CI 95%)
Number of visits	72 (67–77)	57 (54–61)	288 (260–316)	15 (14–16)
Number of diagnosis changes^b^	2.9 (2.7–3.1)	0.9 (0.8–1.0)	10.4 (9.3–11.5)	3.2 (3.0–3.4)
Percentage of visits with a diagnosis change^c^	11.5 (10.4–12.6)	1.9 (1.7–2.1)	8.1 (7.1–9.1)	26.3 (25.3–27.3)
Median time between visits (in days)	156.6 (139.6–173.6)	102.4 (84.9–119.9)	5.5 (4.4–6.6)	300.2 (259.9–340.5)
Number of hospitalizations^d^	11.5 (10.4–12.6)	7.7 (7.0–8.4)	54.9 (48.9–60.9)	1.3 (1.1–1.5)
Number of doctor changes in the trajectory^e^	15.4 (14.2–16.6)	10.8 (10.0–11.6)	63.2 (56.2–70.2)	5.1 (4.8–5.4)
Percentage of visits with a doctor change^f^	27.5 (26.7–28.3)	21.7 (20.7–22.7)	24.9 (23.3–26.5)	37.7 (36.2–39.2)
Percentage of visits with a specialist^g^	61.7 (60.1–63.3)	67.0 (64.9–69.1)	91.3 (89.4–93.2)	42.1 (39.6–44.6)

^a^ Class 1 refers to *Stable diagnosis* trajectory; Class 2 refers to *Unstable diagnosis with high care consumption* trajectory; Class 3 refers to *Intermediate unstable diagnosis with low consumption of care* trajectory.

^b^ Average number of changes in a patient diagnosis occurring between two successive visits along the patient trajectory.

^c^ The number of diagnosis changes divided by the number of visits in the trajectory.

^d^ A hospitalization is defined as a series of visits in a period of 7 days or less.

^e^ The number of times a patient changes from any clinical practitioner to another in two successive visits along the patient trajectory.

^f^ The number of doctor changes divided by the number of visits in the trajectory

^g^ The number of visits performed by a Specialist, as opposed to a General Practitioner, divided by the total number of visits in the trajectory.

The most populous class that we labelled *Stable diagnosis* trajectory, comprised 54% (N = 1258) of all patients. The majority of patients in this class (676 patients; 53.7%) had a predominant diagnosis of SZ, whereas 378 and 204 patients (30% and 16.3%) had a predominant diagnosis of MDP and BD, respectively. Patients had approximately one visit every three months (median time 102 days; CI = 85–120), mostly with psychiatrists (67%; CI = 65–69) and mostly with the same physician (percentage of visits with change of physician 22%; CI = 21–23). Finally, this class was characterized by a low number of changes in diagnosis (0.9; CI = 0.8-1) that occurred at a low frequency across all visits (1.9%; CI = 1.7-2.1), hence the name of the class.

A second class, labelled *Unstable diagnosis with high care consumption* trajectory, contained a smaller number of patients (N = 293; 13%). As in class 1, the majority of patients (N = 227; 77%) had a predominant diagnosis of SZ, and the remaining 23% (N = 66) had a diagnosis of MDD or BD. Individuals in this class had approximately one visit per week, but multiple same-day visits were also observed, suggesting that contacts likely occurred during hospitalizations. Most visits were seen by specialists (91%; CI = 89–93), with a low frequency of change of specialist (percentage of visits with change of physician 25%; CI = 23–27). On average, these individuals had the highest absolute mean number of changes in diagnosis (10.4; CI = 9.3-11.5), but an intermediate frequency of changes in diagnosis when the number of visits was considered (8.1%; CI = 7.1-8.1).

Finally, class 3 labelled *Intermediate unstable diagnosis with low consumption of care* trajectory included 782 patients (34%) mostly affected by mood disorders (N = 636; 81%), whereas 19% (N = 146) had a lifetime diagnosis of schizophrenia. For these patients, visits were fewer (16; CI = 15–17) and more distant in time (about one visit per year) and were less likely to be with a specialist (42% of visits; CI = 40–45%) or with the same physician (percentage of visits with a change of physician 38%; CI = 36–39).

### External validation of the clusters

We conducted a bootstrapping-based stability analysis to assess the robustness of our clustering results based on the method reported in [41]. The procedure involved the following steps: 1. We generated 1,000 resampled datasets through bootstrapping; 2. For each resampled dataset, we applied the same clustering methodology (i.e., hierarchical clustering with Ward’s linkage) to produce the corresponding partitions; 3. To determine a stable partitioning, we employed a majority voting rule, where clusters were assigned based on the most frequently occurring assignments across resamples; 4. We compared the stable partitioning to the original clustering results using the Rand Index [41], which yielded a value of 70.79. This score indicates a strong level of agreement, suggesting that the clustering structure is relatively stable and reproducible.

To further validate the distinction between the three classes, we performed pairwise ANOVAs. One-way ANOVAs were performed with class as the independent variable, resulting in 3 comparisons: Class 1 vs. Class 2, Class 1 vs. Class 3, and Class 2 vs. Class 3. **Table 4** reports the R2 values for each ANOVA, quantifying how much variability in the dependent variable is explained by the independent variable.

**Table 4:** Pairwise ANOVAs with Class of service trajectory as independent variable.

	All three types^a^		1 vs 2^b^		1 vs 3^b^		2 vs 3^b^	
**Characteristic**	**F**	**R** ^**2**^	**F**	**R** ^**2**^	**F**	**R** ^**2**^	**F**	**R** ^**2**^
Number of visits	856	**0.42**	881	**0.36**	344	0.14	971	**0.48**
Number of diagnosis changes^c^	717	**0.38**	1104	**0.42**	708	0.26	362	0.25
Percentage of visits with a diagnosis change^d^	1732	**0.60**	444	0.22	3372	**0.62**	412	0.28
Median time between visits in days	81	0.06	27	0.02	100	0.05	77	0.07
Number of hospitalizations^e^	276	**0.40**	297	**0.35**	90	0.09	701	**0.43**
Number of doctor changes^f^	670	**0.37**	756	**0.33**	122	0.06	696	**0.39**
Percentage of visits with a doctor change^g^	174	0.13	8	0.01	320	0.14	87	0.07
Percentage of visits with a specialist^h^	233	0.17	111	0.07	211	0.09	521	**0.33**

One-way ANOVAs were performed with “class of trajectory” as independent variable considering all three types and pairwise comparisons: Class 1 vs Class 2, Class 1 vs Class 3 and Class 2 vs Class 3. For each ANOVA it is reported **F** and **R**^**2**^. All the measured effects were significant, with p-values lower than 0.001. Values of R^2^ greater than 0.30 highlight the variables best explaining the different trajectory types (in bold).

^a^ degree of freedom = 2.

^b^ degree of freedom = 1.

^c^ The number of different diagnoses in two successive visits along the patient trajectory.

^d^ The number of diagnosis changes divided by the number of visits in the trajectory.

^e^ We define a hospitalization as a series of visits in a period of 7 days or less.

^f^ The number of different doctors in two successive visits along the patient trajectory.

^g^ The number of doctor changes divided by the number of visits in the trajectory.

^h^ The number of visits performed by a Specialist, as opposed to a General Practitioner, divided by the number of visits in the trajectory.

Finally, to test whether the three obtained classes were mainly determined by the service delivery descriptors, the patients’ clinical characteristics and service delivery descriptors were entered as independent variables in a multinomial logistic regression model where the three clusters or classes were used as dependent variables (see **Table 5**). The results indicated that patients’ characteristics such as sex, and predominant diagnosis did not significantly predict the three clusters.

**Table 5:** Multinomial Logistic Regression model with Class of services trajectory as dependent variable.

Independent Variable	OR	95% CI	p-value
Male (reference: Female)	0.71	(0.40–1.25)	0.234
First Diagnosis (reference: BD)			
MDD^a^	0.46	(0.21–1.02)	0.056
SZ^a^	0.58	(0.24–1.41)	0.232
Predominant Diagnosis (reference: BD)			
MDD^a^	1.62	(0.68–3.89)	0.272
SZ^a^	1.56	(0.65–3.81)	0.324
Number of visits	1.03	(1.02–1.05)	**<0.001**
Number of diagnosis changes^b^	1.1	(0.96–1.26)	0.171
Percentage of visits with a diagnosis change^c^	2.23	(2.28–3.80)	**<0.001**
Number of doctor changes^d^	1.00	(0.98–1.02)	0.875
Percentage of visits with a doctor change^e^	5.23	(0.53–52.18)	0.154
Median time between visits in days	1.00	(1.00–1.00)	**0.001**
Number of hospitalizations^f^	1.05	(1.00–1.11)	0.088
Percentage of visits with a specialist^g^	1.17	(0.37–3.66)	0.792

The model has a McFadden R^2^ of 0.89 that, while not exactly correspondent to the classic R^2^, shows that the model explains well the different trajectory types. A bold font highlights the most significative factors.

^a^ BD = Bipolar disorder, MDD = Major depressive disorder, SZ = Schizophrenia.

^b^ The number of different diagnoses in two successive visits along the patient trajectory.

^c^ The number of diagnosis changes divided by the number of visits in the trajectory.

^d^ The number of different doctors in two successive visits along the patient trajectory.

^e^ The number of doctor changes divided by the number of visits in the trajectory.

^f^ We define a hospitalization as a series of visits in a period of 7 days or less.

^g^ The percentage of visits with a specialist is computed as the number of visits performed by a Specialist, as opposed to a General Practitioner, divided by the number of visits in the trajectory.

## Discussion

Our study through cluster analysis revealed three distinct classes of longitudinal service delivery trajectories. The results suggest that these classes apply to patients receiving either a diagnosis of SZ, BD or MDD and that the patients in each class longitudinally use psychiatric resources according to three distinct patterns. The study also supports the relevance of our method for future studies investigating heterogeneity in patients’ long-term trajectories and possibly within-diagnosis heterogeneity.

It is noteworthy that the first trajectory characterizing the largest number of patients featured stable diagnoses, with a low rate of clinician changes and included the majority (around 65%) of the schizophrenia patients. In contrast, the BD patients were more represented in the two other trajectories, showing higher percentages of both diagnoses and doctors changes. Interestingly, these results align with previous studies suggesting that BD diagnosis showed less stability over time than the SZ diagnosis [14–21]. Finally, the second trajectory characterized by unstable diagnosis and a high care consumption that might be associated with hospitalizations, contains only a few of the MDD patients.

The issue of the longitudinal stability of the diagnoses made by the clinicians has received limited attention so far [42]. Our longitudinal analysis suggested that instability of diagnoses and frequent doctor changes across life would be key variables distinguishing subgroups of service trajectories. We believe that these variables should be addressed in future studies since our results showed that 47% of the patients in our sample would fall into the two classes characterized by medium or high rates of diagnosis changes. Furthermore, we found that there was an association between a higher rate of diagnosis changes and a higher rate of physician changes across a patient’s trajectory. However, our study could not distinguish which of the two factors would be causal. In other words, would more demanding or relationally difficult patients provoke more clinician changes or would the changes in clinicians due to the mode of care delivery lead to increased diagnosis changes? Focussing on the determinants and timing of clinician changes is certainly important for future investigations, and previous studies [14–21,43] have already reported trends in diagnosis changes. As regards the practitioner and the patient, our findings also call to mind the potential disadvantages of discontinuities in care for the patient care and for cost efficiency, as well as consequences for patient or family perceptions of illness, and even forensic implications since the nature of diagnosis of a psychiatric disorder has an influence in the court of law. Meanwhile, there would generally be advantages, in the management of delivery of psychiatric care, of paying more attention to the rate of practitioner changes for these patients who in a large proportion have a chronic course.

Two other results of our analysis may have general implications for health care planners/managers and clinicians. First, MDD episodes may often be the start of a trajectory toward a lifetime predominant diagnosis of either MDD, BD or SZ. In particular, 1014 of the 2333 patients in our sample received a first diagnosis of MDD, but MDD seems to be the predominant diagnosis for only 759 of them. Second, the three trajectories show significant differences in the way patients with same diagnosis would longitudinally interact with the care delivery system. A logical consequence would be that diagnosis in the first episodes of illness might not be the best indicator for planning the provision of personalized mental health services.

Our study has both strengths and limitations. Several limitations merit cautions in the interpretation of the results. A *first* one to consider is that we analyzed longitudinal data collected from 2002 to 2014 and the possibility of a cohort effect might be kept in mind when interpreting the results, although there is no evidence of such an operative cohort effect in Quebec’s universal health care system. *Second*, whereas the stability of the cluster structure was confirmed by a statistical bootstrapping, we did not practice an out-of-sample validation. *Third*, our data could not allow us to determine the extent to which the patients themselves initiated their interactions with the care delivery system or vice-versa. In other words, it is for instance possible that some patients’ lower compliance placed them on the unstable diagnostic trajectory associated with low service use*. Fourth*, the type and number of clinical variables defining disease specificity or severity may also limit the interpretation. *Fifth,* the use of clinician diagnosis in governmental database must be kept in mind regarding potential differences with research diagnoses [11]. *Sixth*, the non-availability of the pharmacological history could have given valuable information that might have influenced the results.

Several strengths of our study should also be highlighted. *First*, this study is, to the best of our knowledge, the first to examine long-term psychiatric patient trajectories that incorporate service delivery descriptors. Our findings suggest that the latter may contribute to the distinction among subgroups of trajectories. *Second,* governmental database studies imply diagnoses made by clinicians. The use of ICD diagnoses renders our results readily useful for non-scientist clinical practitioners and furthermore, may allow other research groups to reproduce our results in governmental databases from other countries, paving the way for testing their own service descriptors. *Third,* the results of the cluster analysis align with those produced by classical regression models, thereby supporting the validity of the service trajectory classes produced by the clustering analytical methods. *Fourth*, the cluster analysis conducted in our study may present an advantage over other data-driven approaches that require thorough modeling among model variables.

## Conclusion

Schizophrenia (SZ), bipolar disorder (BD), and major depressive disorder (MDD) exhibit intra-diagnostic heterogeneity in their clinical courses, which poses significant challenges for the planning of appropriate service delivery to these patients. This paper presents a clustering-based method that integrates service delivery and patient clinical descriptors to study the long-term trajectories of patients affected by SZ, BD, or MDD. Applied to a sample of 2,333 patients, the method identified three distinct classes or groups of patients that interact differently with the service provider in terms of the number and the frequency of the visits and demonstrated varying levels of diagnostic instability. These results suggest that analyzing service delivery features may be valuable for gaining further insights into a patient’s long-term clinical heterogeneity. Incorporating the pharmacological history into such studies would undoubtedly provide additional benefits.

## Supporting information

S1 Table Frequency of diagnosis changes among Major Depressive Disorder (MDD), Bipolar Disorder (BD), and Schizophrenia (SZ) over the total diagnosis changes. (DOCX)

S2 Table Demographic and clinical characteristics of female patients (N = 1068) and each service trajectory class. (DOCX)

S3 Table Average values and confidence intervals at 95% for the service trajectories measures of female patients (N = 1068) and each service trajectory class. (DOCX)

S4 Table Demographic and clinical characteristics of male patients (N = 1265) and each service trajectory class. (DOCX)

S5 Table Average values and confidence intervals at 95% for the service trajectories measures of male patients and each service trajectory class. (DOCX)

S6 Table Demographic and clinical characteristics of patients with a predominant diagnosis of Schizophrenia (N = 1049) and each service trajectory class. (DOCX)

S7 Table Average values and confidence intervals at 95% for service trajectories measures of patients with a predominant diagnosis of Schizophrenia (N = 1049) and each service trajectory class. (DOCX)

S8 Table Demographic and clinical characteristics of patients with a predominant diagnosis of Bipolar disorder (N = 1049) and each service trajectory class. (DOCX)

S9 Table Average values and confidence intervals at 95% for service trajectories measures of patients with a predominant diagnosis of Bipolar disorder (N = 525) and each service trajectory class. (DOCX)

S10 Table Demographic and clinical characteristics of patients with a predominant diagnosis of Major Depressive Disorder (N = 759) and each service trajectory class. (DOCX)

S11 Table Average values and confidence intervals at 95% for service trajectories measures of patients with a predominant diagnosis of Major Depressive Disorder (N = 759)) and each service trajectory class. (DOCX)

S12 Table ICD-09 diagnostic codes used to classify our patients in the three disorder categories. (DOCX)

S1 Fig Correlation matrix. Correlation coefficients of the five clustering variables are reported and shaded in blue for positive correlation and in red for negative correlation. A more intense color corresponds to a stronger correlation.
(DOCX)

S2 Fig Distribution densities. For each clustering variable, kernel density estimates are proposed: a) visit frequency; b) median time between consecutive visits; c) number of diagnosis changes; d) percentage of visits with a diagnosis change; e) number of physician changes.
(DOCX)
