# Supplementary material for: Analyzing service descriptors and patients’ clinical characteristics may help understand heterogeneity in long-term trajectory of patients with schizophrenia, bipolar and major depressive disorder
Source: PLOS Ment Health. 2025 May 14;2(5):e0000327. doi: 10.1371/journal.pmen.0000327 (PMC12798446; doi:10.1371/journal.pmen.0000327)
Supplement: S1 Table — (DOCX) [file pmen.0000327.s001.docx]

**S1 Table. Frequency of diagnosis changes among Major Depressive Disorder (MDD), Bipolar Disorder (BD), and Schizophrenia (SZ) over the total diagnosis changes.**

| **Type of change** | **Final Sample N (%)** |
| --- | --- |
| From MDD to BD | 1321 (19.6%) |
| From MDD to SZ | 1297 (19.2%) |
| From BD to MDD | 1243 (18.4%) |
| From BD to SZ | 859 (13.3%) |
| From SZ to MDD | 1156 (17.1%) |
| From SZ to BD | 839 (12.4%) |

Number of diagnosis changes between diseases are reported in absolute number of changes, and in percentage over the total number of diagnosis changes. Patients with several changes may appear in multiple rows.
