# Supplementary material for: Analyzing service descriptors and patients’ clinical characteristics may help understand heterogeneity in long-term trajectory of patients with schizophrenia, bipolar and major depressive disorder
Source: PLOS Ment Health. 2025 May 14;2(5):e0000327. doi: 10.1371/journal.pmen.0000327 (PMC12798446; doi:10.1371/journal.pmen.0000327)
Supplement: S2 Table — (DOCX) [file pmen.0000327.s002.docx]

**S2 Table. Demographic and clinical characteristics of female patients (N=1068) and each service trajectory class^a^**

| **Demographic and clinical characteristics** | **Female patients** |  | **Class 1** |  | **Class 2** |  | **Class 3** |
| --- | --- | --- | --- | --- | --- | --- | --- |
|  | **N (%)** |  | **N (%)** |  | **N (%)** |  | **N (%)** |
| Female patients | 1068 (100%) |  | 260 (24%) |  | 543 (51%) |  | 265 (25%) |
| Patients with a first diagnosis of: |  |  |  |  |  |  |  |
| *Major Depressive Disorder* | 620 (58%) |  | 182 (70%) |  | 280 (52%) |  | 158 (60%) |
| *Bipolar Disorder* | 241 (23%) |  | 43 (16%) |  | 116 (21%) |  | 82 (31%) |
| *Schizophrenia* | 207 (19%) |  | 35 (14%) |  | 147 (57%) |  | 25 (9%) |
| Patients with a predominant diagnosis of: |  |  |  |  |  |  |  |
| *Major Depressive Disorder* | 498 (47%) |  | 182 (70%) |  | 177 (33%) |  | 139 (53%) |
| *Bipolar Disorder* | 300 (28%) |  | 43 (16%) |  | 158 (29%) |  | 99 (37%) |
| *Schizophrenia* | 270 (25%) |  | 35 (14%) |  | 208 (38%) |  | 27 (10%) |

^a^ Class 1 refers to *Stable diagnosis* trajectory; Class 2 refers to *Unstable diagnosis with high care consumption* trajectory; Class 3 refers to *Intermediate unstable diagnosis with low consumption of care* trajectory.
