# Supplementary material for: Analyzing service descriptors and patients’ clinical characteristics may help understand heterogeneity in long-term trajectory of patients with schizophrenia, bipolar and major depressive disorder
Source: PLOS Ment Health. 2025 May 14;2(5):e0000327. doi: 10.1371/journal.pmen.0000327 (PMC12798446; doi:10.1371/journal.pmen.0000327)
Supplement: S3 Table — (DOCX) [file pmen.0000327.s003.docx]

**S3 Table. Average values and confidence intervals at 95% for the service trajectories measures of female patients (N=1068) and each service trajectory class^a^**

|  |  | **Female patients** |  | **Class 1** |  | **Class 2** |  | **Class 3** |
| --- | --- | --- | --- | --- | --- | --- | --- | --- |
| **Characteristics** |  | **(CI 95%)** |  | **(CI 95%)** |  | **(CI 95%)** |  | **(CI 95%)** |
| Number of visits |  | 55.3  (49.0–61.6) |  | 19.6  (17.3–21.9) |  | 92.4  (81.0–103.8) |  | 14.5  (12.4–16.6) |
| Number of diagnosis changes^b^ |  | 3.3  (3.0–3.6) |  | 0 |  | 4.4  (3.9–4.9) |  | 4.3  (3.7–4.9) |
| Percentage of visits with a diagnosis change^c^ |  | 12.6  (11.7–13.5) |  | 0 |  | 8.1  (7.6–8.6) |  | 34.0  (32.5–35.5) |
| Median time between visits (in days) |  | 168.6  (142.5–194.7) |  | 237.7  (174.1–301.3) |  | 31.5  (27.0–36.0) |  | 381.7  (305.2–458.2) |
| Number of hospitalizations^d^ |  | 7.9  (6.6–9.2) |  | 1.3  (1.0–1.6) |  | 14.4  (12.1–16.7) |  | 1.3  (0.9–1.7) |
| Number of doctor changes in the trajectory^e^ |  | 11.8  (10.3–13.3) |  | 3.1  (2.7–3.5) |  | 19.1  (16.2–22.0) |  | 5.5  (4.7–6.3) |
| Percentage of visits with a doctor change^f^ |  | 27.3  (26.1–28.5) |  | 24.4  (21.6–27.2) |  | 22%  (20.8–23.2) |  | 41  (38.3–43.7) |
| Percentage of visits with a specialist^g^ |  | 53.2  (50.8–55.6) |  | 37.6  (32.7–42.5) |  | 68.0  (64.9–71.1) |  | 38.2  (34.2–42.2) |

^a^ Class 1 refers to *Stable diagnosis* trajectory; Class 2 refers to *Unstable diagnosis with high care consumption* trajectory; Class 3 refers to *Intermediate unstable diagnosis with low consumption of care* trajectory.

^b^ The mean number of changes in a patient diagnosis occurring between two successive visits along the patient trajectory.

^c^ The number of diagnosis changes divided by the number of visits in the trajectory.

^d^ A hospitalization is defined as a series of visits in a period of time of 7 days or less.

^e^ The number of times when a patient changes from any clinical practitioner to another in two successive visits along the patient trajectory.

^f^ Computed as the number of doctor changes divided by the number of visits in the trajectory

^g^ Computed as the number of visits performed by a Specialist, as opposed to a General Practitioner, divided by the total number of visits in the trajectory.
