# Supplementary material for: Analyzing service descriptors and patients’ clinical characteristics may help understand heterogeneity in long-term trajectory of patients with schizophrenia, bipolar and major depressive disorder
Source: PLOS Ment Health. 2025 May 14;2(5):e0000327. doi: 10.1371/journal.pmen.0000327 (PMC12798446; doi:10.1371/journal.pmen.0000327)
Supplement: S4 Table — (DOCX) [file pmen.0000327.s004.docx]

**S4 Table. Demographic and clinical characteristics of male patients (N=1265) and each service trajectory class^a^**

| **Demographic and clinical characteristics** | **Male patients** |  | **Class 1** |  | **Class 2** |  | **Class 3** |
| --- | --- | --- | --- | --- | --- | --- | --- |
|  | **N (%)** |  | **N (%)** |  | **N (%)** |  | **N (%)** |
| Male patients | 1265 (100%) |  | 116 (9%) |  | 920 (73%) |  | 229 (18%) |
| Patients with a first diagnosis of: |  |  |  |  |  |  |  |
| *Major Depressive Disorder* | 394 (31%) |  | 10 (9%) |  | 285 (31%) |  | 99 (43%) |
| *Bipolar Disorder* | 232 (18%) |  | 9 (8%) |  | 149 (16%) |  | 74 (32%) |
| *Schizophrenia* | 639 (51%) |  | 97 (83%) |  | 486 (53%) |  | 56 (25%) |
| Patients with a predominant diagnosis of: |  |  |  |  |  |  |  |
| *Major Depressive Disorder* | 261 (21%) |  | 0 (0%) |  | 170 (18%) |  | 91 (40%) |
| *Bipolar Disorder* | 225 (18%) |  | 1 (1%) |  | 145 (16%) |  | 79 (34%) |
| *Schizophrenia* | 779 (61%) |  | 115 (99%) |  | 605 (66%) |  | 59 (26%) |

^a^ Class 1 refers to *Stable diagnosis* trajectory; Class 2 refers to *Unstable diagnosis with high care consumption* trajectory; Class 3 refers to *Intermediate unstable diagnosis with low consumption of care* trajectory.
