# Supplementary material for: Analyzing service descriptors and patients’ clinical characteristics may help understand heterogeneity in long-term trajectory of patients with schizophrenia, bipolar and major depressive disorder
Source: PLOS Ment Health. 2025 May 14;2(5):e0000327. doi: 10.1371/journal.pmen.0000327 (PMC12798446; doi:10.1371/journal.pmen.0000327)
Supplement: S5 Table — (DOCX) [file pmen.0000327.s005.docx]

**S5 Table. Average values and confidence intervals at 95% for the service trajectories measures of male patients and each service trajectory class^a^**

|  |  | **Male patients** |  | **Class 1** |  | **Class 2** |  | **Class 3** |
| --- | --- | --- | --- | --- | --- | --- | --- | --- |
| **Characteristics** |  | **(CI 95%)** |  | **(CI 95%)** |  | **(CI 95%)** |  | **(CI 95%)** |
| Number of visits |  | 87.1  (79.1–95.1) |  | 450.1  (409.2–491) |  | 59.9  (55.9–63.9) |  | 12.9  (11.3–14.5) |
| Number of diagnosis changes^b^ |  | 2.5  (2.3–2.7) |  | 2.5  (1.6–3.4) |  | 2.3  (2–2.6) |  | 3.7  (3.3–4.1) |
| Percentage of visits with a diagnosis change^c^ |  | 9.4  (8.6–10.2) |  | 0.5  (0.3–0.7) |  | 4.4  (4–4.8) |  | 34.3  (32.5–36.1) |
| Median time between visits (in days) |  | 146.4  (124–168.8) |  | 1.9  (1.7–2.1) |  | 78.7  (63.4–94) |  | 491.6  (395.7–587.5) |
| Number of hospitalizations^d^ |  | 14.5  (12.8–16.2) |  | 91.2  (82.4–100) |  | 8.2  (7.4–9) |  | 1.0  (0.8–1.2) |
| Number of doctor changes in the trajectory^e^ |  | 18.5  (16.7–20.3) |  | 89.9  (77.8–102) |  | 12.8  (11.7–13.9) |  | 5.4  (4.6–6.2) |
| Percentage of visits with a doctor change^f^ |  | 27.6  (26.5–28.7) |  | 19.9  (18.2–21.6) |  | 24  (22.8–25.2) |  | 46.1  (43.2–49) |
| Percentage of visits with a specialist^g^ |  | 68.9  (66.9–70.9) |  | 98.4  (97.8–99) |  | 72.8  (70.5–75.1) |  | 38.2  (33.8–42.6) |

^a^ Class 1 refers to *Stable diagnosis* trajectory; Class 2 refers to *Unstable diagnosis with high care consumption* trajectory; Class 3 refers to *Intermediate unstable diagnosis with low consumption of care* trajectory.

^b^ The mean number of changes in a patient diagnosis occurring between two successive visits along the patient trajectory.

^c^ The number of diagnosis changes divided by the number of visits in the trajectory.

^d^ A hospitalization is defined as a series of visits in a period of time of 7 days or less.

^e^ The number of times when a patient changes from any clinical practitioner to another in two successive visits along the patient trajectory.

^f^ The number of doctor changes divided by the number of visits in the trajectory

^g^ The number of visits performed by a Specialist, as opposed to a General Practitioner, divided by the total number of visits in the trajectory.
