# Supplementary material for: Analyzing service descriptors and patients’ clinical characteristics may help understand heterogeneity in long-term trajectory of patients with schizophrenia, bipolar and major depressive disorder
Source: PLOS Ment Health. 2025 May 14;2(5):e0000327. doi: 10.1371/journal.pmen.0000327 (PMC12798446; doi:10.1371/journal.pmen.0000327)
Supplement: S6 Table — (DOCX) [file pmen.0000327.s006.docx]

**S6 Table. Demographic and clinical characteristics of patients with a predominant diagnosis of Schizophrenia (N=1049) and each service trajectory class^a^**

| **Demographic and clinical characteristics** | **SZ patients** |  | **Class 1** |  | **Class 2** |  | **Class 3** |
| --- | --- | --- | --- | --- | --- | --- | --- |
|  | **N (%)** |  | **N (%)** |  | **N (%)** |  | **N (%)** |
| Male patients | 779 (74%) |  | 416 (82%) |  | 174 (73%) |  | 189 (63%) |
| Patients with a first diagnosis of: |  |  |  |  |  |  |  |
| *Major Depressive Disorder* | 201 (19%) |  | 54 (11%) |  | 34 (14%) |  | 113 (38%) |
| *Bipolar Disorder* | 84 (8%) |  | 22 (4%) |  | 27 (11%) |  | 35 (12%) |
| *Schizophrenia* | 764 (73%) |  | 434 (85%) |  | 179 (75%) |  | 151 (50%) |
| Patients with a predominant diagnosis of: |  |  |  |  |  |  |  |
| *Major Depressive Disorder* | 0 (0%) |  | 0 (0%) |  | 0 (0%) |  | 0 (0%) |
| *Bipolar Disorder* | 0 (0%) |  | 0 (0%) |  | 0 (0%) |  | 0 (0%) |
| *Schizophrenia* | 1049 (100%) |  | 510 (100%) |  | 240 (100%) |  | 299 (100%) |

^a^ Class 1 refers to *Stable diagnosis* trajectory; Class 2 refers to *Unstable diagnosis with high care consumption* trajectory; Class 3 refers to *Intermediate unstable diagnosis with low consumption of care* trajectory.
