# Supplementary material for: Analyzing service descriptors and patients’ clinical characteristics may help understand heterogeneity in long-term trajectory of patients with schizophrenia, bipolar and major depressive disorder
Source: PLOS Ment Health. 2025 May 14;2(5):e0000327. doi: 10.1371/journal.pmen.0000327 (PMC12798446; doi:10.1371/journal.pmen.0000327)
Supplement: S7 Table — (DOCX) [file pmen.0000327.s007.docx]

**S7 Table. Average values and confidence intervals at 95% for service trajectories measures of patients with a predominant diagnosis of Schizophrenia (N=1049) and each service trajectory class^a^**

|  |  | **SZ patients** |  | **Class 1** |  | **Class 2** |  | **Class 3** |
| --- | --- | --- | --- | --- | --- | --- | --- | --- |
| **Characteristics** |  | **N**  **(CI 95%)** |  | **N**  **(CI 95%)** |  | **N**  **(CI 95%)** |  | **N**  **(CI 95%)** |
| Number of visits |  | 127.8  (117.5, 138.1) |  | 65.0  (60.7, 69.3) |  | 341.9  (313.6, 370.2) |  | 62.9  (51.6, 74.2) |
| Number of diagnosis changes^b^ |  | 3.2  (2.8, 3.6) |  | 0.5  (0.4, 0.6) |  | 3.6  (3, 4.2) |  | 7.5  (6.5, 8.5) |
| Percentage of visits with a diagnosis change^c^ |  | 5.5  (4.9, 6.1) |  | 0.8  (0.7, 0.9) |  | 1.6  (1.3, 1.9) |  | 16.6  (15.3, 17.9) |
| Median time between visits (in days) |  | 48.4  (37.1, 59.7) |  | 66.1  (45.9, 86.3) |  | 2.6  (2.3, 2.9) |  | 54.9  (36, 73.8) |
| Number of hospitalizations^d^ |  | 22.2  (20.1, 24.3) |  | 8.8  (8, 9.6) |  | 66.4  (60.2, 72.6) |  | 9.7  (7.6, 11.8) |
| Number of doctor changes in the trajectory^e^ |  | 27.2  (24.7, 29.7) |  | 12.1  (11.1, 13.1) |  | 70.9  (63, 78.8) |  | 18.0  (15.2, 20.8) |
| Percentage of visits with a doctor change^f^ |  | 24.6  (23.6, 25.6) |  | 20.9  (19.4, 22.4) |  | 21.0  (19.7, 22.3) |  | 33.7  (31.7, 35.7) |
| Percentage of visits with a specialist^g^ |  | 85.3  (83.8, 86.8) |  | 85.5  (83.3, 87.7) |  | 96.8  (95.9, 97.7) |  | 75.7  (72.6, 78.8) |

^a^ Class 1 refers to *Stable diagnosis* trajectory; Class 2 refers to *Unstable diagnosis with high care consumption* trajectory; Class 3 refers to *Intermediate unstable diagnosis with low consumption of care* trajectory.

^b^ The mean number of changes in a patient diagnosis occurring between two successive visits along the patient trajectory.

^c^ The number of diagnosis changes divided by the number of visits in the trajectory.

^d^ A hospitalization is defined as a series of visits in a period of time of 7 days or less.

^e^ The number of times when a patient changes from any clinical practitioner to another in two successive visits along the patient trajectory.

^f^ The number of doctor changes divided by the number of visits in the trajectory

^g^ The number of visits performed by a Specialist, as opposed to a General Practitioner, divided by the total number of visits in the trajectory.
