# Supplementary material for: Analyzing service descriptors and patients’ clinical characteristics may help understand heterogeneity in long-term trajectory of patients with schizophrenia, bipolar and major depressive disorder
Source: PLOS Ment Health. 2025 May 14;2(5):e0000327. doi: 10.1371/journal.pmen.0000327 (PMC12798446; doi:10.1371/journal.pmen.0000327)
Supplement: S8 Table — (DOCX) [file pmen.0000327.s008.docx]

**S8 Table. Demographic and clinical characteristics of patients with a predominant diagnosis of Bipolar disorder (N=1049) and each service trajectory class^a^**

| **Demographic and clinical characteristics** | **BD patients** |  | **Class 1** |  | **Class 2** |  | **Class 3** |
| --- | --- | --- | --- | --- | --- | --- | --- |
|  | **N (%)** |  | **N (%)** |  | **N (%)** |  | **N (%)** |
| Male patients | 225 (43%) |  | 76 (41%) |  | 127 (44%) |  | 22 (41%) |
| Patients with a first diagnosis of: |  |  |  |  |  |  |  |
| *Major Depressive Disorder* | 194 (37%) |  | 53 (29%) |  | 124 (43%) |  | 17 (31%) |
| *Bipolar Disorder* | 297 (57%) |  | 126 (68%) |  | 137 (48%) |  | 34 (63%) |
| *Schizophrenia* | 34 (6%) |  | 6 (3%) |  | 25 (9%) |  | 3 (6%) |
| Patients with a predominant diagnosis of: |  |  |  |  |  |  |  |
| *Major Depressive Disorder* | 0 (0%) |  | 0 (0%) |  | 0 (0%) |  | 0 (0%) |
| *Bipolar Disorder* | 525 (100%) |  | 185 (100%) |  | 286 (100%) |  | 54 (100%) |
| *Schizophrenia* | 0 (0%) |  | 0 (0%) |  | 0 (0%) |  | 0 (0%) |

^a^ Class 1 refers to *Stable diagnosis* trajectory; Class 2 refers to *Unstable diagnosis with high care consumption* trajectory; Class 3 refers to *Intermediate unstable diagnosis with low consumption of care* trajectory.
