# Supplementary material for: Analyzing service descriptors and patients’ clinical characteristics may help understand heterogeneity in long-term trajectory of patients with schizophrenia, bipolar and major depressive disorder
Source: PLOS Ment Health. 2025 May 14;2(5):e0000327. doi: 10.1371/journal.pmen.0000327 (PMC12798446; doi:10.1371/journal.pmen.0000327)
Supplement: S9 Table — (DOCX) [file pmen.0000327.s009.docx]

**S1 Table 9. Average values and confidence intervals at 95% for service trajectories measures of patients with a predominant diagnosis of Bipolar disorder (N=525) and each service trajectory class^a^**

|  |  | **BD patients** |  | **Class 1** |  | **Class 2** |  | **Class 3** |
| --- | --- | --- | --- | --- | --- | --- | --- | --- |
| **Characteristics** |  | **(CI 95%)** |  | **(CI 95%)** |  | **(CI 95%)** |  | **(CI 95%)** |
| Number of visits |  | 35.2  (30.3, 40.1) |  | 31.0  (27.6, 34.4) |  | 43.7  (35.1, 52.3) |  | 4.8  (3.7, 5.9) |
| Number of diagnosis changes^b^ |  | 3.6  (3.2, 4) |  | 1.1  (0.9, 1.3) |  | 5.5  (4.9, 6.1) |  | 2.6  (2, 3.2) |
| Percentage of visits with a diagnosis change^c^ |  | 17.1  (15.7, 18.5) |  | 3.0  (2.5, 3.5) |  | 19.2  (18.1, 20.3) |  | 53.9  (52.6, 55.2) |
| Median time between visits (in days) |  | 252.9  (206, 299.8) |  | 153.5  (105.5, 201.5) |  | 137.9  (105.3, 170.5) |  | 1202.1  (911.7, 1492.5) |
| Number of hospitalizations^d^ |  | 4.0  (3, 5) |  | 2.4  (1.9, 2.9) |  | 5.7  (4, 7.4) |  | 0.1  (0, 0.2) |
| Number of doctor changes in the trajectory^e^ |  | 7.1  (6.2, 8) |  | 4.6  (3.8, 5.4) |  | 9.6  (8.2, 11) |  | 2.3  (1.6, 3) |
| Percentage of visits with a doctor change^f^ |  | 27.5  (25.7, 29.3) |  | 17.9  (15.3, 20.5) |  | 29.8  (27.5, 32.1) |  | 47.6  (41, 54.2) |
| Percentage of visits with a specialist^g^ |  | 56.6  (53.3, 59.9) |  | 62.3  (56.7, 67.9) |  | 58.5  (54.2, 62.8) |  | 27.1  (18.5, 35.7) |

^a^ Class 1 refers to *Stable diagnosis* trajectory; Class 2 refers to *Unstable diagnosis with high care consumption* trajectory; Class 3 refers to *Intermediate unstable diagnosis with low consumption of care* trajectory.

^b^ The mean number of changes in a patient diagnosis occurring between two successive visits along the patient trajectory.

^c^ The number of diagnosis changes divided by the number of visits in the trajectory.

^d^ A hospitalization is defined as a series of visits in a period of time of 7 days or less.

^e^ The number of times when a patient changes from any clinical practitioner to another in two successive visits along the patient trajectory.

^f^ The number of doctor changes divided by the number of visits in the trajectory

^g^ The number of visits performed by a Specialist, as opposed to a General Practitioner, divided by the total number of visits in the trajectory.
