# Supplementary material for: Analyzing service descriptors and patients’ clinical characteristics may help understand heterogeneity in long-term trajectory of patients with schizophrenia, bipolar and major depressive disorder
Source: PLOS Ment Health. 2025 May 14;2(5):e0000327. doi: 10.1371/journal.pmen.0000327 (PMC12798446; doi:10.1371/journal.pmen.0000327)
Supplement: S10 Table — (DOCX) [file pmen.0000327.s010.docx]

**S10 Table. Demographic and clinical characteristics of patients with a predominant diagnosis of Major Depressive Disorder (N=759) and each service trajectory class^a^**

| **Demographic and clinical characteristics** | **MDD patients** |  | **Class 1** |  | **Class 2** |  | **Class 3** |
| --- | --- | --- | --- | --- | --- | --- | --- |
|  | **N (%)** |  | **N (%)** |  | **N (%)** |  | **N (%)** |
| Male patients | 261 (34%) |  | 81 (28%) |  | 129 (35%) |  | 51 (49%) |
| Patients with a first diagnosis of: |  |  |  |  |  |  |  |
| *Major Depressive Disorder* | 619 (82%) |  | 281 (99%) |  | 276 (75%) |  | 62 (59%) |
| *Bipolar Disorder* | 92 (12%) |  | 3 (1%) |  | 63 (17%) |  | 26 (25%) |
| *Schizophrenia* | 48 (6%) |  | 1 (0%) |  | 30 (8%) |  | 17 (16%) |
| Patients with a predominant diagnosis of: |  |  |  |  |  |  |  |
| *Major Depressive Disorder* | 759 (100%) |  | 285 (100%) |  | 369 (100%) |  | 105 (100%) |
| *Bipolar Disorder* | 0 (0%) |  | 0 (0%) |  | 0 (0%) |  | 0 (0%) |
| *Schizophrenia* | 0 (0%) |  | 0 (0%) |  | 0 (0%) |  | 0 (0%) |

^a^ Class 1 refers to *Stable diagnosis* trajectory; Class 2 refers to *Unstable diagnosis with high care consumption* trajectory; Class 3 refers to *Intermediate unstable diagnosis with low consumption of care* trajectory.
