# Supplementary material for: Analyzing service descriptors and patients’ clinical characteristics may help understand heterogeneity in long-term trajectory of patients with schizophrenia, bipolar and major depressive disorder
Source: PLOS Ment Health. 2025 May 14;2(5):e0000327. doi: 10.1371/journal.pmen.0000327 (PMC12798446; doi:10.1371/journal.pmen.0000327)
Supplement: S11 Table — (DOCX) [file pmen.0000327.s011.docx]

**S11 Table. Average values and confidence intervals at 95% for service trajectories measures of patients with a predominant diagnosis of Major Depressive Disorder (N=759)) and each service trajectory class^a^**

|  |  | **MDD patients** |  | **Class 1** |  | **Class 2** |  | **Class 3** |
| --- | --- | --- | --- | --- | --- | --- | --- | --- |
| **Characteristics** |  | **(CI 95%)** |  | **(CI 95%)** |  | **(CI 95%)** |  | **(CI 95%)** |
| Number of visits |  | 22.2  (19.7, 24.7) |  | 25.5  (20.3, 30.7) |  | 24.6  (21.7, 27.5) |  | 4.7  (4.2, 5.2) |
| Number of diagnosis changes^b^ |  | 1.9  (1.7, 2.1) |  | 0.1  (0, 0.2) |  | 3.3  (3, 3.6) |  | 2.2  (1.9, 2.5) |
| Percentage of visits with a diagnosis change^c^ |  | 14.0  (12.9, 15.1) |  | 0.2  (0.1, 0.3) |  | 15.8  (15, 16.6) |  | 45.1  (43, 47.2) |
| Median time between visits (in days) |  | 239.4  (203.3, 275.5) |  | 216.4  (157.9, 274.9) |  | 77.3  (62.4, 92.2) |  | 871.6  (719.9, 1023.3) |
| Number of hospitalizations^d^ |  | 1.9  (1.4, 2.4) |  | 2.2  (1.1, 3.3) |  | 2.1  (1.6, 2.6) |  | 0.2  (0.1, 0.3) |
| Number of doctor changes in the trajectory^e^ |  | 5.0  (4.5, 5.5) |  | 4.2  (3.5, 4.9) |  | 6.3  (5.4, 7.2) |  | 2.4  (2, 2.8) |
| Percentage of visits with a doctor change^f^ |  | 31.5  (29.9, 33.1) |  | 25.8  (23.2, 28.4) |  | 31  (28.8, 33.2) |  | 48.8  (44.4, 53.2) |
| Percentage of visits with a specialist^g^ |  | 32.6  (30.1, 35.1) |  | 29.8  (25.7, 33.9) |  | 36.6  (33.1, 40.1) |  | 26.1  (20.4, 31.8) |

^a^ Class 1 refers to *Stable diagnosis* trajectory; Class 2 refers to *Unstable diagnosis with high care consumption* trajectory; Class 3 refers to *Intermediate unstable diagnosis with low consumption of care* trajectory.

^b^ The mean number of changes in a patient diagnosis occurring between two successive visits along the patient trajectory.

^c^ The number of diagnosis changes divided by the number of visits in the trajectory.

^d^ A hospitalization is defined as a series of visits in a period of time of 7 days or less.

^e^ The number of times when a patient changes from any clinical practitioner to another in two successive visits along the patient trajectory.

^f^ The number of doctor changes divided by the number of visits in the trajectory

^g^ The number of visits performed by a Specialist, as opposed to a General Practitioner, divided by the total number of visits in the trajectory.
