# Supplementary material for: Analyzing service descriptors and patients’ clinical characteristics may help understand heterogeneity in long-term trajectory of patients with schizophrenia, bipolar and major depressive disorder
Source: PLOS Ment Health. 2025 May 14;2(5):e0000327. doi: 10.1371/journal.pmen.0000327 (PMC12798446; doi:10.1371/journal.pmen.0000327)
Supplement: S12 Table — (DOCX) [file pmen.0000327.s012.docx]

**S12 Table.** **ICD-09 diagnostic codes used to classify our patients in the three disorder categories**

| **Major Depressive Disorder** | | **Bipolar Disorder** | | **Schizophrenia** | |
| --- | --- | --- | --- | --- | --- |
| 2962 | Major depressive disorder single episode | 2960 | Bipolar I disorder, single manic episode | 295 | Schizophrenic disorders |
| 2963 | Major depressive disorder recurrent episode | 2964 | Bipolar I disorder, most recent episode (or current) manic | 2950 | Simple type |
| 311 | Depressive disorder, not elsewhere classified | 2965 | Bipolar I disorder, most recent episode (or current) depressed | 2951 | Disorganized type |
|  |  | 2966 | Bipolar I disorder, most recent episode (or current) mixed | 2952 | Catatonic type |
|  |  | 2967 | Bipolar I disorder, most recent episode (or current) unspecified | 2953 | Paranoid type |
|  |  | 2968 | Other and unspecified bipolar disorders | 2954 | Schizophreniform disorder |
|  |  |  |  | 2955 | Latent schizophrenia |
|  |  |  |  | 2956 | Schizophrenic disorder, residual type |
|  |  |  |  | 2957 | Schizoaffective disorder |
|  |  |  |  | 2958 | Other specified types |
|  |  |  |  | 2959 | Unspecified schizophrenia |
