# Supplementary material for: Analyzing service descriptors and patients’ clinical characteristics may help understand heterogeneity in long-term trajectory of patients with schizophrenia, bipolar and major depressive disorder
Source: PLOS Ment Health. 2025 May 14;2(5):e0000327. doi: 10.1371/journal.pmen.0000327 (PMC12798446; doi:10.1371/journal.pmen.0000327)
Supplement: S1 Fig — Correlation coefficients of the five clustering variables are reported and shaded in blue for positive correlation and in red for negative correlation. A more intense color corresponds to a stronger correlation. (DOCX) [file pmen.0000327.s013.docx]

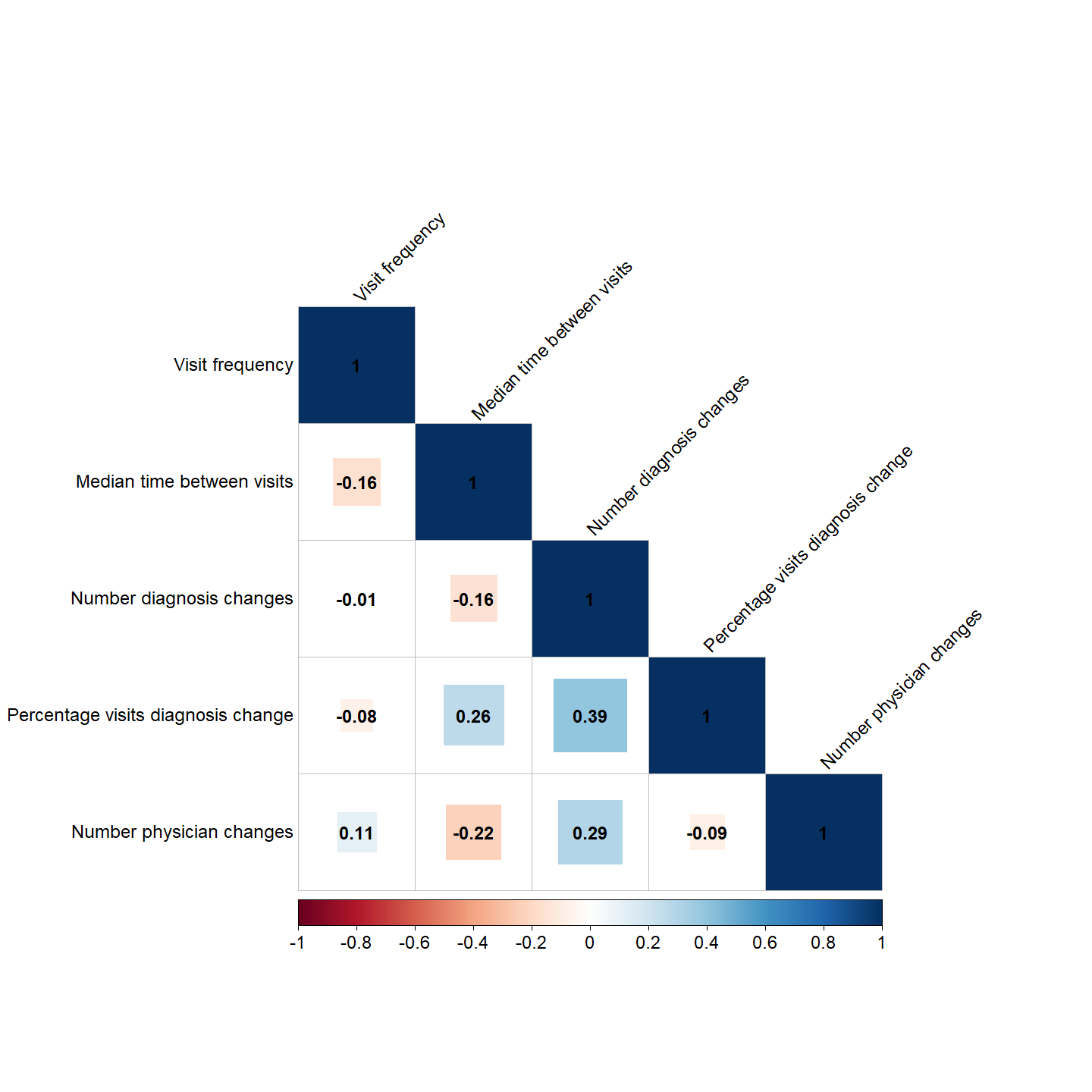


**S1 Fig.** **Correlation matrix.** Correlation coefficients of the five clustering variables are reported and shaded in blue for positive correlation and in red for negative correlation. A more intense color corresponds to a stronger correlation.
