# Supplementary material for: Analyzing service descriptors and patients’ clinical characteristics may help understand heterogeneity in long-term trajectory of patients with schizophrenia, bipolar and major depressive disorder
Source: PLOS Ment Health. 2025 May 14;2(5):e0000327. doi: 10.1371/journal.pmen.0000327 (PMC12798446; doi:10.1371/journal.pmen.0000327)
Supplement: S2 Fig — For each clustering variable, kernel density estimates are proposed: a) visit frequency; b) median time between consecutive visits; c) number of diagnosis changes; d) percentage of visits with a diagnosis change; e) number of physician changes. (DOCX) [file pmen.0000327.s014.docx]

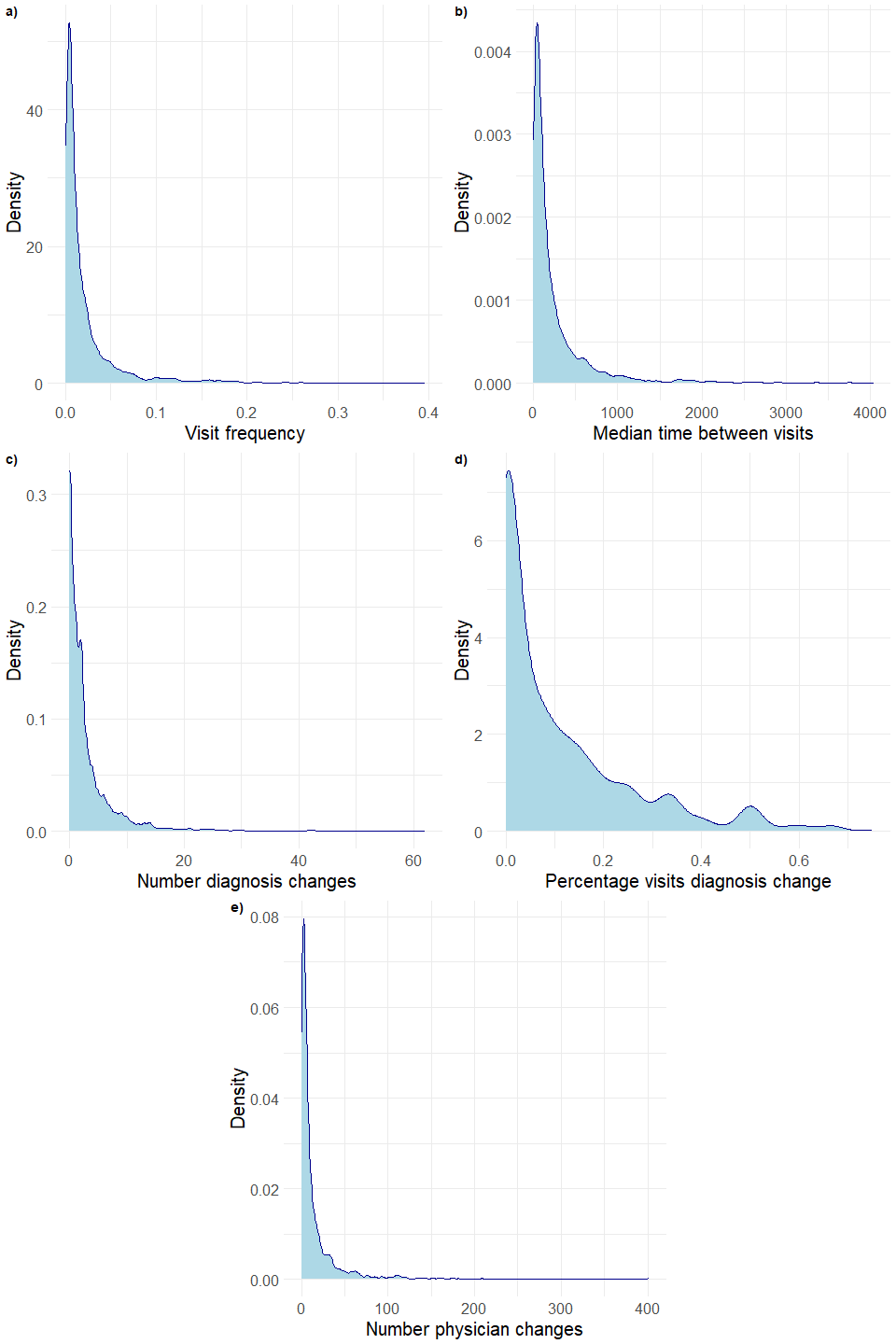


**S2 Fig.** **Distribution densities.** For each clustering variable, kernel density estimates are proposed: a) visit frequency; b) median time between consecutive visits; c) number of diagnosis changes; d) percentage of visits with a diagnosis change; e) number of physician changes.
